# Single object profiles regression analysis (SOPRA): a novel method for analyzing high-content cell-based screens

**DOI:** 10.1186/s12859-022-04981-8

**Published:** 2022-10-21

**Authors:** Rajendra Kumar Gurumurthy, Klaus-Peter Pleissner, Cindrilla Chumduri, Thomas F. Meyer, André P. Mäurer

**Affiliations:** 1 Department of Molecular Biology, Max Planck Institute for Infection Biology, 10117 Berlin, Germany; 2 Steinbeis Center for Systems Biomedicine, Steinbeis Innovation gGmbH, 14612 Falkensee, Germany

**Keywords:** Image analysis, Cell-based assay, Loss-of-function screen, Drug screening, RNA interference analysis

## Abstract

**Background:**

High-content screening (HCS) experiments generate complex data from multiple object features for each cell within a treated population. Usually, these data are analyzed by using population-averaged values of the features of interest, increasing the amount of false positives and the need for intensive follow-up validation. Therefore, there is a strong need for novel approaches with reproducible hit prediction by identifying significantly altered cell populations.

**Results:**

Here we describe SOPRA, a workflow for analyzing image-based HCS data based on regression analysis of non-averaged object features from cell populations, which can be run on hundreds of samples using different cell features. Following plate-wise normalization, the values are counted within predetermined binning intervals, generating unique frequency distribution profiles (histograms) for each population, which are then normalized to control populations (control-based normalization). These control-normalized frequency distribution profiles are analyzed using the Bioconductor R-package maSigPro, originally developed to analyze time profiles. However, statistically significant altered frequency distributions are also identified by maSigPro when integrating it into the SOPRA workflow. Finally, significantly changed profiles can be used to generate a heatmap from which altered cell populations with similar phenotypes can be identified, enabling the detection of siRNAs and compounds with the same ‘on-target’ profile and reducing the number of false positive hits.

**Conclusions:**

SOPRA is a novel analysis workflow for the detection of statistically significant normalized frequency distribution profiles of cellular features generated in high-throughput RNAi screens. For the validation of the SOPRA software workflow, a screen for cell cycle progression was used. We were able to identify such profiles for siRNA-mediated gene perturbations and chemical inhibitors of different cell cycle stages. The SOPRA software is freely available from Github.

**Supplementary Information:**

The online version contains supplementary material available at 10.1186/s12859-022-04981-8.

## Background

The availability of robotic liquid handling combined with automated fluorescence microscopy and high-performance image computing has enabled rapid advances in developing high-throughput screening [1]. Numerous studies have demonstrated the power of high-throughput image-based assays for characterizing drug effects [2], identifying active small molecules [3], and classifying sub-cellular protein localization [4, 5], including genome-wide siRNA-mediated loss-of-function screens [6] or gene deletion libraries [7]. For every single cell within a cellular sample population, it is possible to achieve quantitative measurements of phenotypes such as expression level and localization of proteins, post-translational modifications, and even cellular or sub-cellular morphologies.

Analyzing cellular populations in the early drug discovery process allows the complexity of living systems to be addressed and produces vast amounts of data that are more meaningful than those obtained from isolated proteins [8]. With advanced bioinformatics tools, treatments can be identified that lead to altered cell populations and, therefore, might be relevant drugs or drug targets.

Nonetheless, several limitations in data analysis have restricted the full potential of high-throughput image-based assays so far [9, 10]. The usual course of events for an HCS analysis workflow starts with extracting image feature data, followed by normalization and statistical analysis, including final hit selection [11]. A wide variety of microscopes, image analysis, and data analysis software packages are available to address these issues [12]. However, distributions of multidimensional, multivariate phenotypic measurements from cellular populations are mostly transformed into single population-averaged values such as mean or median values. These population-averaged values are used for plate-wise or batch-wise normalizations, as well as for statistical analysis for hit selection [13, 14], which leads to a substantial loss of information. Population-averaged values can indicate whether the value of the measured phenotype increases or decreases upon treatment but do not reflect the detailed response of a cellular population to a certain treatment or gene depletion. Therefore, these population-averaged values are limiting the power of the statistical approaches that are widely used, such as Z-Score or percent-of-control (POC) analysis, making it impossible to identify more distinct reactions of a cell population. This loss of information also hampers the differentiation of treatments or gene depletions with the same ‘on-target’ effect from those with ‘off-target’ effects, which is extremely important for RNAi gene perturbation experiments, where multiple siRNAs are used per gene.

Some publications have described methods for non-averaged cell population data analysis from high-content image-based screens. Knapp et al. [15] showed considerable effects of population context on observed phenotypes when using non-averaged population data for the normalization steps but still used population-averaged values for hit detection. Another method uses multivariate cell classification based on phenotypic changes for hit identification [16], which results in a drug effect score, and a vector, indicating the simultaneous phenotypic changes induced by the drug. Another publication used multi-parametric phenotypic profiles to cluster genes based on morphological changes in individual cells [17]. Yet another group has proposed using Ripley’s K-function to identify knockdowns resulting in perturbation of this cell clustering [18]. Also, the Kolomogorow-Smirnov (KS) test has been used to score the difference between control and sample populations [19]. However, all these methods have limitations that prevent them from widely used for large-scale high-content cell population analysis. Multivariate classification methods are mainly based on analyzing predominantly redundant image features. Spatial clustering requires a subjective and work intensive classification step for the cellular populations, whereas KS only uses one unique value to identify cell populations with altered distributions.

Here we present a new approach called Single Object Profiles Regression Analysis (SOPRA) that overcomes many limitations by analyzing non-averaged cell population data. It uses classification-free regression analysis of normalized (control-based) frequency distribution profiles of cell populations. SOPRA can be used to analyze data derived from various high-throughput techniques, such as images from automated microscopy or single-cell data from FACS analysis. The regression workflow consists of (i) a pre-processing step, (ii) data gathering and normalization steps, (iii) identifying significant profiles, (iv) post-processing. The normalization is performed in a plate-wise and bin-wise fashion, resulting in a unique normalized frequency distribution profile for each feature of a cell population. Finally, normalized frequency distribution profiles exhibiting statistically significant changes are identified using a *p*-value and R-squared (RSQ)-value derived from the regression analysis with the R-package maSigPro [20, 21]. Additionally, normalized profiles of various cell features identified as significantly altered can be further clustered in a heatmap according to their similarity. This can be used to identify treatments with the same ‘on-target’ effects. Most loss-of-function screens use multiple siRNAs for the same gene, which should end up in the same cluster if they have a similar cell population phenotype. The more siRNAs for the same gene are identified as having a similar cell population profile, the more reliably this gene can be regarded as a hit. Beyond this, the derived values of a regression analysis of frequency distribution profiles of cellular features are not affected by experimental bias to the same degree as population-averaged approaches [22], leading to more reproducible results. We used a cell-based chemical compound and RNAi screen of cell cycle progression to validating the SOPRA workflow. The cellular features ‘Area’, ‘Total Intensity DAPI’, and ‘Mean Intensity DAPI’ were extracted for each nucleus using image analysis software and subjected to the SOPRA workflow. We found that SOPRA can be used to identify statistically significant changes in frequency distribution profiles within cellular populations, whether induced by gene perturbation through siRNAs or by chemical inhibitor treatment. Taken together, SOPRA is a novel object-based data analysis workflow based on regression analysis of distribution profiles of cellular features to identify significantly changed cell populations from high-throughput data sets.

## Results

**Fig. 1:**
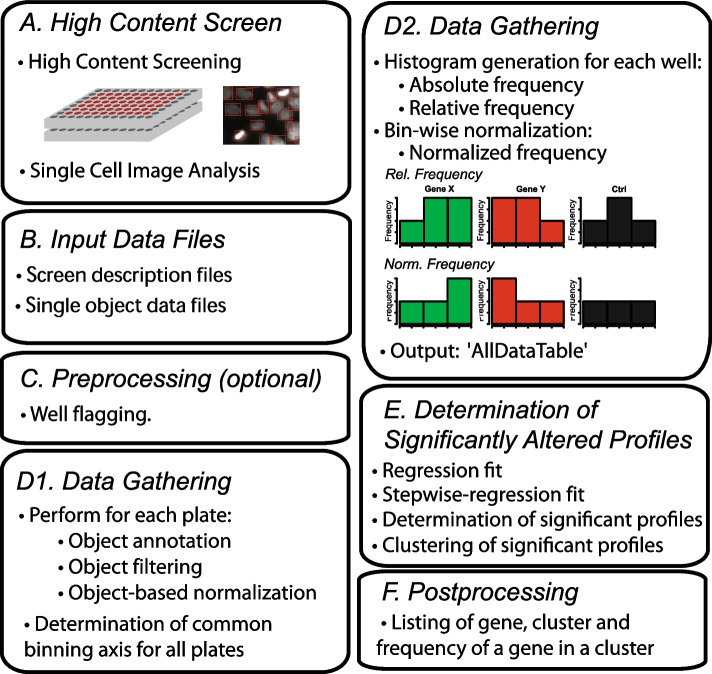
SOPRA workflow of high-throughput data sets. **A** High content screening data is generated and used to prepare single object data files and input data files. **B** Screen description and the single cell data files are generated manually. **C** Wells that should be omitted are flagged, and **D1** the single object data is filtered, normalized to the median of the controls, and a common binning axis for all plates is determined. **D2** For each measured feature, the frequency distribution profile (histogram) is generated for each sample well, which is then normalized for each bin to the median distribution profile of the controls. **E** Significantly changed normalized distribution profiles are determined using regression analysis, and **F** a post-processing step is performed to determine the number of screening hits

SOPRA utilizes a data gathering step combined with plate-wise and so-called bin-wise normalization methods, as well as a two-step regression approach that first adjusts a global regression model with defined variables in order to identify profiles exhibiting statistically significant changes [20] (Conesa et al., 2006). The SOPRA workflow consists of several steps, as outlined in Fig. 1. *A: High Content Screen.* This first step includes screening, image analysis, and data extraction. B: Preparation of screen description files and the single cell data files. *C: Preprocessing (optional).* The derived data files for various image features at the single cell level are subjected to a pre-processing step to exclude all data from flagged wells that should be excluded from the analysis. *D1: Data Gathering and Plate-Wise Normalization*. In this step, each single cell object is annotated with additional information, such as RNA.ID, plate number, well number, replicate number, well content, and gene symbol. If the imaging software supports a gating procedure for objects that do not meet certain criteria, such as cell size, these can also be flagged and excluded from subsequent analysis steps. The measured value of each cell for the feature of interest is then normalized to the median of the objects in the neutral control wells. *D2: Data Gathering and Frequency Distribution Profiles (Histogram) Generation.* Next, the common binning axis of the distribution profiles is generated by determining the minimum and maximum limits of the measured feature across all the screen data to avoid strong relative differences at the tails of the distribution. The data are divided into equally spaced binning intervals, sufficient for population data that follows a given order of regression model (such as quadratic). A pseudo count of one is added to each bin to avoid bins with zero objects. First, the absolute frequency distribution is computed, and then the relative frequency for each treatment is calculated by dividing the number of objects in each bin by the population size (sum of all objects in all bins). Next, a bin-wise normalization step is performed by dividing the relative frequency of each bin for each treatment by the median of the corresponding bin of the control wells, such as the ‘AllStars’ control. That means that the frequency density distribution (histogram) of a selected feature (test distribution) is divided by the frequency distribution (histogram) of that selected feature for control wells (reference distribution) in a bin-wise manner (control-based normalization). The resulting profiles can be analyzed (using the generated bins instead of time points) in the R-package maSigPro [21]. Taking, for instance, the logarithm of the ratio test/reference one gets a zero profile if the test distribution equals the reference distribution. The larger the deviation of profiles from the reference distribution, the more likely the test distribution would be statistically significant. Finally, an AllDataTable.txt-file containing all normalized and annotated data is generated. This AllDataTable.txt-file serves as input for determining statistically significant different histogram profiles using maSigPro.

Binning creates equal-length bins to which data are assigned. The default number n of bins (the binning level) is 7.

For variable x, assume that the data set is *{x*_*i*_*},* let *x*_*1*_*, x*_*2*_*,….x*_*m*_ represent the ordered values of the variable. Let the xth percentile be *min(x)* and *max(x)*. The range of the variable is *range(x)* = *max(x) – min(x)*. For binning, the width of binning interval is$L=\frac{max\left(x\right) - min\left(x\right)}{n}$. The split points are *s*_*k*_ = *min(x)* + L * k, where *k* = *1, 2,…, numbin-1* and *numbin* is *n*. For each bin a pseudocount of 1 is added *Countp(X*_*ik*_*)* = *Count(X*_*ik*_*)* + *1*.

*E: Determination of Statistically Significant Altered Profiles*. Regression analysis is performed using the Bioconductor R-package maSigPro [21] to identify significantly altered normalized distribution profiles. *F: Postprocessing (optional).* The gene, cluster, and gene frequency within the cluster are listed for all significantly changed normalized distribution profiles identified by the maSigPro analysis.

### Data generation of cell cycle progression screen

**Fig. 2:**
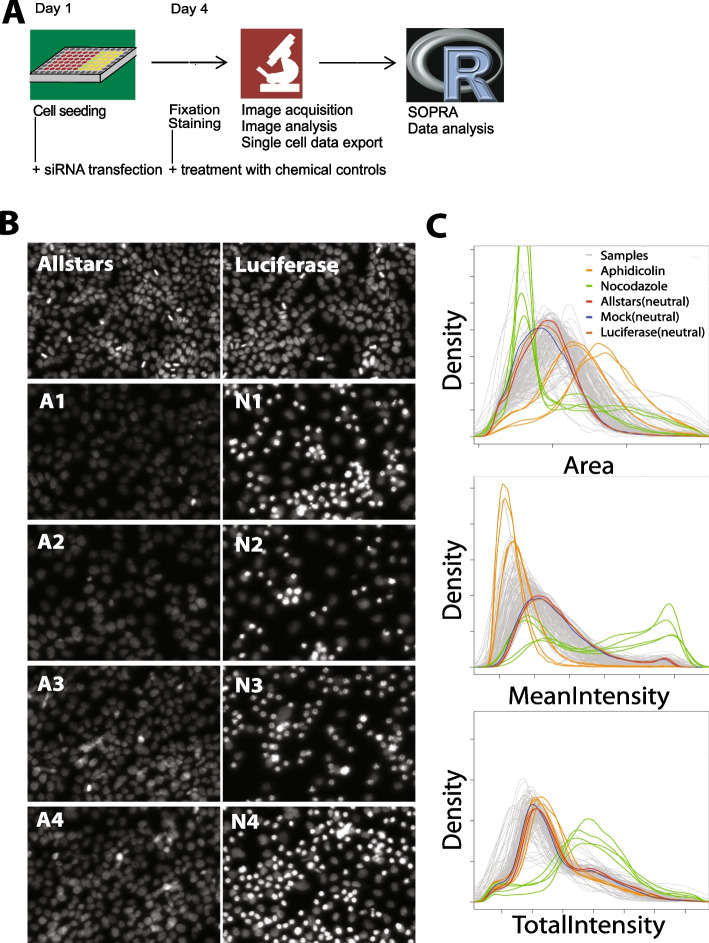
Schematic representation of the microscopic cell cycle screening assay. **A** Cells were seeded in 384-well plates and treated with siRNAs or chemical cell cycle inhibitors at different concentrations and time points to inhibit cell cycle progression. Cells were fixed, stained with Hoechst, and subjected to automated microscopy and image analysis. **B** Treatment with the control-siRNAs AllStars and luciferase did not lead to any cell population changes. Treatment with aphidocoline A1 (2 µg/ml, 24 h), A2 (4 µg/ml, 24 h), A3 (2 µg/ml, 12 h), A4 (4 µg/ml, 12 h) and nocodazole N1 (50 ng/ml, 24 h), N2 (75 ng/ml, 24 h), N3 (50 ng/ml, 12 h), N4 (75 ng/ml, 12 h) resulted in cell populations arrested at various stages of the cell cycle. **C** Distribution profiles were generated for each well from the data exported for the features ‘Area’, ‘Mean Intensity DAPI’ and ‘Total Intensity DAPI’ for all nuclei

To generate the cell cycle progression screen data, we seeded HeLa cells in 384-well plates either transfected or treated with the inhibitors in three independent biological replicates (Fig. 2A). We used 166 different siRNAs to target 107 genes, from which 54 had been reported to interfere with cell cycle progression [23] (Additional file 1: Fig. 1, Additional file 2: Table 1). Additionally, we used cells treated with the chemical inhibitors aphidicolin or nocodazole, which lead to G1/S and G2/M cell cycle arrest, respectively. Cells left untreated (Mock) or treated with siRNAs against ‘AllStars’ or ‘Luciferase’ were used as negative controls. While images from AllStars- and Luciferase-treated cell populations showed an unaltered, normal phenotype, treatment of cells with aphidicolin (A1-4) or nocodazole (N1-4) resulted in an altered phenotype as a consequence of G1/S or G2/M phase arrest, respectively (Fig. 2B). On day 4, cells were fixed, nuclei stained with Hoechst (Fig. 2A), images acquired using automated microscopy, and automated image analysis (ScanR: High-Content Screening Station for Life Science, Olympus) was performed for extracting the image features ‘Area’, ‘Total Intensity DAPI’ and ‘Mean Intensity DAPI’ for each nucleus (Additional file 3: Fig. 2) in tab-delimited files using a ScanR export script. The cell population distribution profiles for the control as well as the chemically or siRNA-treated samples, behave differently for the extracted object features (Fig. 2C). They show a strong shift towards smaller nuclei for nocodazole and towards larger nuclei for aphidicolin-treated samples for the feature ‘Area’, while for the feature ‘Mean Intensity DAPI’ the influence of these two chemical treatments on the mean intensity is the opposite. Interestingly, for the feature ‘Total Intensity DAPI’ a strong shift towards higher values was observed for nocodazole-treated samples, while aphidicolin treatment did not alter the profile compared to that of the ‘Allstars’, ‘Luciferase’ or ‘Mock’-treated wells. Distribution profiles of the cell populations treated with different siRNAs (samples) showed no clear tendency (Fig. 2C).

**Fig. 3:**
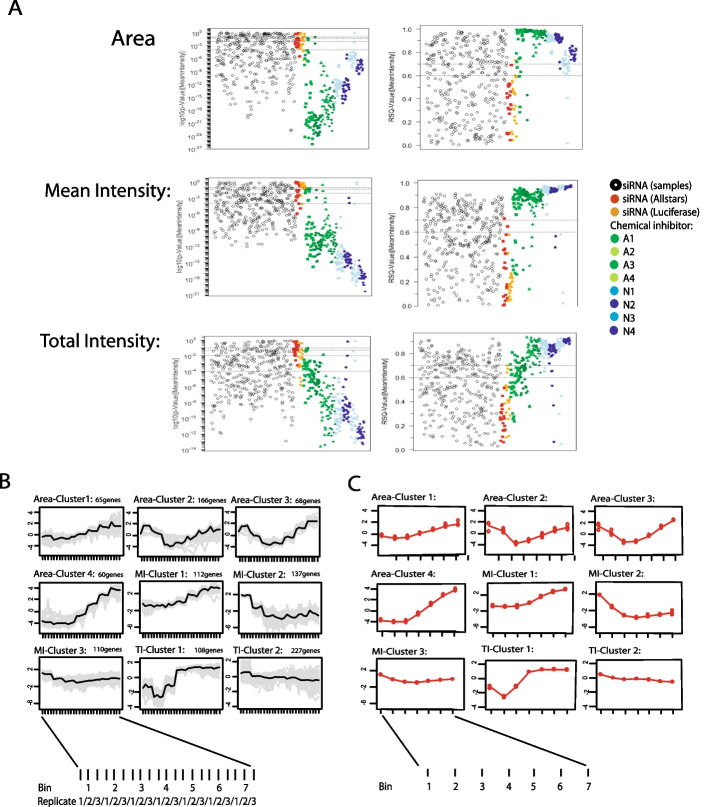
Results of SOPRA regression analysis and cluster profiles. **A** Calculated RSQ and *p*-values of each well for the features ‘Area’, ‘Mean Intensity DAPI’, and ‘Total Intensity DAPI’ using the maSigPro package. **B** Data visualization by cluster analysis. Normalized distribution profiles of all significantly altered normalized profiles for the three image features were clustered using k-means with 4, 3 and 2 clusters, respectively. The average feature profile is shown (black line) together with the individual profiles of the cell populations in the cluster (grey lines) or **C** as the mean of 3 replicates

**Fig. 4:**
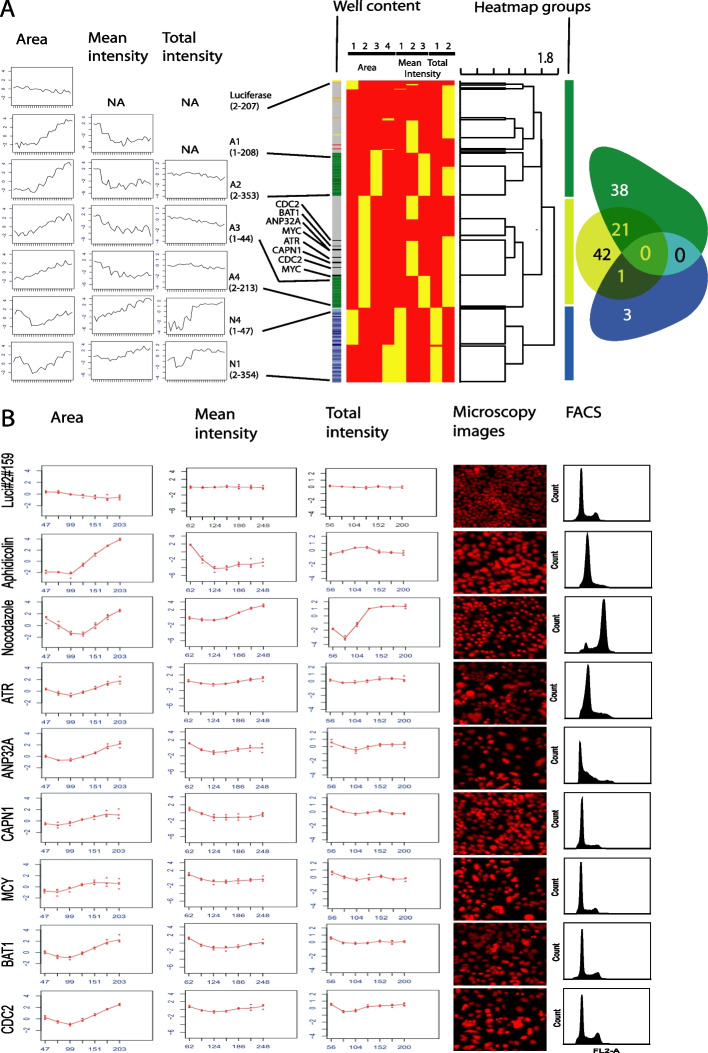
Heatmap analysis and examples of significantly altered distribution profiles. **A** The normalized regression profiles for different treatment conditions for aphidicolin (A1–A4) and nocodazole (N1 and N4), as well as Luciferase are displayed. A heatmap showed the distribution of all cell populations with at least one significantly changed profile for the features 'Area', 'Mean Intensity DAPI' and 'Total Intensity DAPI' among the SOPRA cluster profiles. Wells treated with aphidicolin or nocodazole are displayed in shades of green or blue in the row sidebar. Wells Mock-treated or treated with siRNA against Luciferase or AllStars are indicated in red, orange and yellow, respectively. Wells treated with siRNA against specific genes are displayed in grey in the row sidebar. The heatmap is clustered using hierarchical clustering, and a dendrogram with a cut-off of 1.8 is performed, resulting in the heatmap groups (1), (2) and (3). The Venn diagram displays the distribution of the significantly changed profiles for each treatment among the heatmap groups (1–3). **B** Examples of profiles for the features ‘Area’, ‘Total Intensity DAPI’ and ‘Mean Intensity DAPI’ of cell populations significantly changed upon siRNA treatment, as well as the corresponding microscopic and FACS images

We calculated *p*-values and RSQ-values using maSigPro regression analysis, as described, to identify significantly altered distribution profiles compared to the neutral controls. The maSigPro package computes a regression fit for each frequency distribution profile and uses a linear step-up (BH) false discovery rate (FDR) procedure [24]. Here, we used a level of 0.05 for FDR control. Once statistically significant distribution profiles have been found, a variable selection procedure is applied to find significant variables for each profile. The final step is to generate lists of statistically significant profiles. As expected, cell populations treated with the ‘AllStars’ or ‘Luciferase’ controls usually had high *p*-values and low RSQ- values. Only two (10%) and four (20%) out of 20 cellular populations treated with the neutral controls ‘Allstars’ or ‘Luciferase’, respectively, were identified to be significantly changed for at least one of the three cellular features used (Additional file 4: Table 2; Fig. 4A—Plate2, Well 207). When hits were only considered positive if at least two of the image features were identified as significantly changed, none of the neutral controls were identified as a hit. In contrast, cells treated with aphidicoline (A1-4) or nocodazole (N1-4) showed significant changes, indicated by low p-values and high RSQ-values for all of the three extracted cellular features (Fig. 3A). All 28 profiles for each of the aphidicolin conditions A2 (4 µg/ml/24 h), A3 (2 µg/ml/12 h) and A4 (4 µg/ml/12 h), for each of the nocodazole conditions N1 (50 ng/ml/24 h), N2 (75 ng/ml/24 h), N3 (50 ng/ml/12 h) and N4 (75 ng/ml/12 h) and 27 out of 28 profiles for the aphidicolin condition A1 (2 µg/ml/24 h) were identified as significantly changed hits (Additional file 5: Fig. 3). Interestingly, aphidicolin-treated samples showed marked differences for the cellular features ‘Area’ and ‘Mean Intensity’ and only slight changes for the cell feature ‘Total Intensity DAPI’ (Fig. 4A – A1: Plate1, Well 208, A2: Plate2, Well 353, A3: Plate1, Well 44, A4: Plate2, Well 213), while nocodazole-treated samples showed strong changes in all three cellular features used (Fig. 4A -N4: Plate1, Well 47, N1: Plate 2, Well 354). In total, using these thresholds for the *p*-value and the RSQ-value, 359 normalized distribution profiles were identified as significantly altered for each of the cellular features ‘Area’ and ‘Mean Intensity DAPI’ and 335 normalized distribution profiles for the cellular feature ‘Total Intensity DAPI’. This resulted in a total of 448 significantly changed cell populations, with 247 profiles significantly changed for all three, 111 profiles for two, and 90 profiles for only one of the analyzed cellular features. Next, for the 448 profiles identified as significantly changed, a k-means clustering approach was performed (Fig. 3B and Fig. 3C). The normalized distribution profiles for the features ‘Area’, ‘Mean Intensity DAPI’, and ‘Total Intensity DAPI’ were arranged in four, three, and two profile clusters, respectively. Cluster numbers were selected to give high cluster reproducibility. Finally, for all clustered profiles, a heatmap was defined based on the k-means clustering result arranged as a vector (consisting of zeros and ones such as 0001–010-10 for a profile resulting in cluster 4 for ‘Area’, cluster 2 for ‘Mean Intensity DAPI’ and cluster 1 for ‘Total Intensity DAPI’). The heatmap was sorted using a hierarchical clustering (hclust) algorithm to identify cell populations with similar distribution profiles (Fig. 4A). Finally, a dendrogram cut-off value of 1.8 was used to generate three main groups in the matrix.

As a result, the aphidicolin-treated samples A1 and A2 grouped differently from the aphidicolin-treated samples A3 and A4, (Fig. 3A, sidebar), while the nocodazole-treated samples N1 and N2, as well as N3 and N4, grouped together. Further, the significant distribution profiles of samples treated with siRNA were more dispersed in the heatmap, depending on the individual feature distribution. Thus, with this two-step method—first identifying statistically significant normalized profiles for each analyzed image feature, then using a heatmap to generate profile groups – we were able to differentiate cell populations showing a similar distribution among the cluster profiles. Taken together, the SOPRA workflow was responsive enough to distinguish not only nocodazole-treated from aphidicolin-treated samples, but also to differentiate between samples that were treated with the same concentration but for different durations (A1/A2 vs. A3/A4).

As laid out above, analyzed features for siRNAs that target the same gene and have the same ‘on-target’ phenotype should end up in the same cluster and also in the same heatmap group. Therefore, we further analyzed if individual siRNAs for the same gene were represented in the same or different heatmap groups. The individual siRNAs of 38 and 42 genes appeared exclusively in profile groups 1 or 2, respectively, strengthening their ‘on-target’ specificity. In contrast, both groups represented individual siRNAs of 21 genes, indicating less stringent ‘on-target’ specificity or other influences, such as experimental variation. For the SOPRA workflow, two different siRNAs were used for each gene in duplicate, therefore, hits were classified as medium or weak hits if the two siRNAs did not show the same cluster profile and were not grouped in the same heatmap group.

**Fig. 5:**
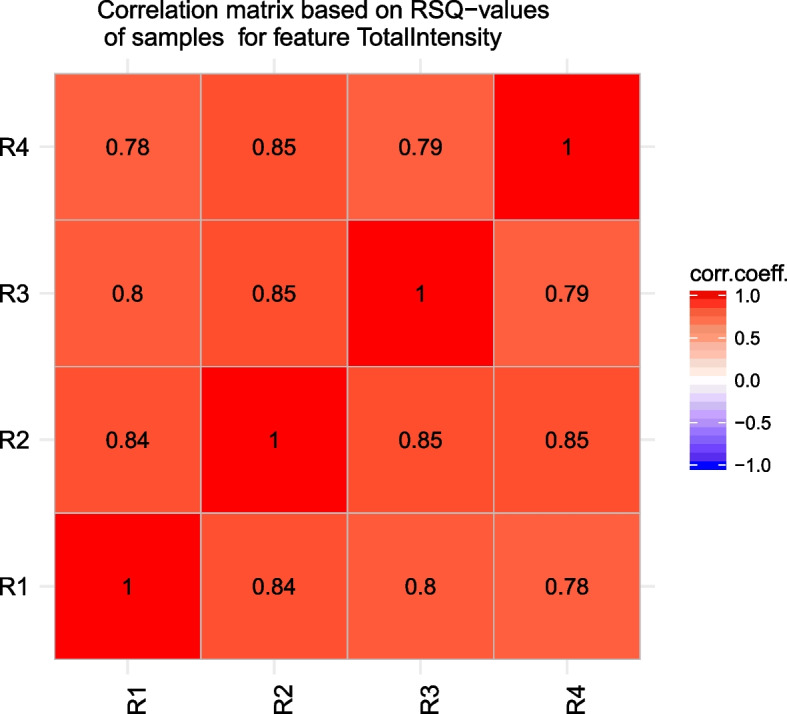
Reproducibility assessment between replicates. Correlation matrix between the RSQ-values for sample data from the different replicate groups R1- R4 for cell feature ‘Total Intensity DAPI’

To assess the reproducibility of plate replicates and SOPRA workflow, the RSQ-values for different groups of replicates were determined and the correlation matrix between these groups was calculated. Firstly, we defined replicate group R1 containing replicate r2, r3, and r4 (i.e., without replicate 1), replicate group R2 containing r1, r3, and r4 (without replicate 2) and so on. Running SOPRA for cell feature ‘Total Intensity DAPI’ with pre-defined maSigPro parameters alfa = 1, *Q* = 1, and RSQ = 0 one gets the *p*-values and RSQ-values for each replicate group. The correlation matrix between the RSQ-values for sample data from the different groups R1–R4 is calculated and visualized (Fig. 5).

**Fig. 6:**
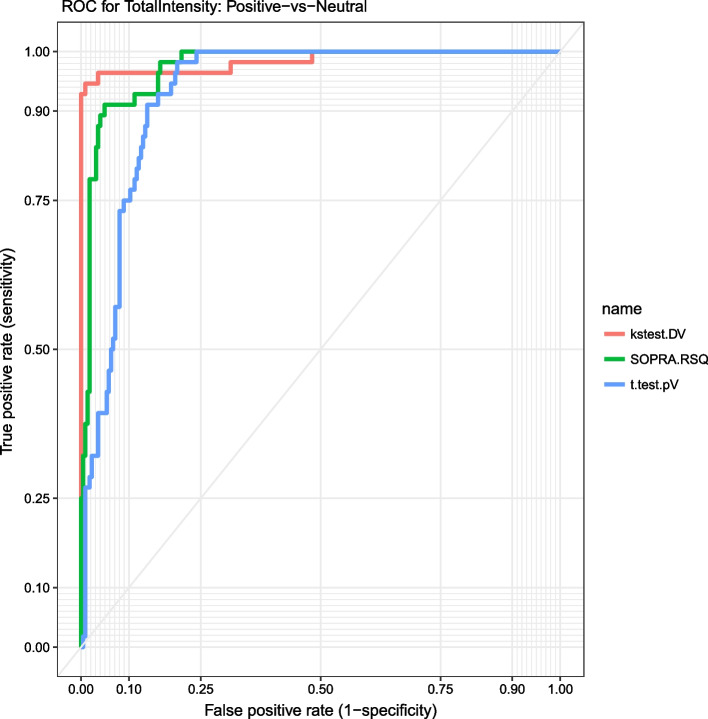
Assessment of diagnostic quality by Reciever Operating Curve (ROC). Receiver Operating Characteristic (ROC) serves to assess the SOPRA workflow in comparison to other statistical approaches, such as “t.test” and “Kolmogorov–Smirnov (KS) test”. The RO curves for cell feature ‘Total Intensity DAPI’ shows that the SOPRA method lies between the t.test and KS-test

Furthermore, we used Receiver Operating Characteristic (ROC) to assess the statistical performance of SOPRA workflow in comparison to other approaches, such as “Kolmogorov–Smirnov (KS) test” which uses probability density and “t.test” for assessment of population differences. The RO curve for cell feature ‘Total Intensity DAPI’ is depicted in Fig. 6 and shows that the SOPRA method lies between the other two RO curves.

To benchmark the efficiency of this method in gene perturbation hit prediction, we tested whether the results of the SOPRA workflow could be validated by either the original cell cycle data from Kittler et al*.* [23] or FACS data generated by our group (Additional file 5: Fig. 3B). We selected 46 of the genes (hits and non-hits) analyzed with SOPRA and performed FACS analysis for cell cycle profiles with one siRNA per gene. A hit was scored as positive for a particular method if at least one other method also leads to the same (positive or negative) result. Out of the 46 genes analyzed, 30 genes from the Kittler et al*.* study were validated with at least one of the other methods (SOPRA or FACS), while for the SOPRA and FACS analyses, 36 and 38 genes, respectively, were validated by one of the other two methods. Taken together, SOPRA and FACS analysis scored best in their ability to predict hits compared to the data published by Kittler et al. [23].

Thus, the SOPRA workflow offers a unique and fast analysis approach based on measured single features of cell populations, comparable to or better than published methods. In contrast to FACS data analysis, it does not need manual intervention or thresholding, such as cell gating. SOPRA is, therefore, well suited for high-throughput and high-content data, as it can be easily run on multiple features from an identical cell population.

In order to prove the scalability, we leveraged the SOPRA software workflow to analyze a genome-wide NFKB screen consisting of 192 384-wells plates with 3 replicates for each plate, i.e., in total 576 plates. Because one plate takes about 50–60 s depending on the operating system, CPU time of the computer model, and disk drives in the network, the SOPRA workflow successfully finished after 60*576 = 34,560 s or 9.60 h provided a large enough R memory.

## Conclusions

Most methods published for analyzing high-content microscopic screens use population-averaged values or manually performed cell classification steps for normalization and hit classification. The SOPRA workflow represents a novel approach for analyzing large microscopy-based high-content screens using non-averaged data of cell populations for normalization and hit determination. The workflow generates frequency distribution profiles of cellular features normalized to a neutral control for each treatment. These normalized distribution profiles are used for hit identification by regression analysis to identify significantly altered profiles using the R-package maSigPro, as originally described for the analysis of single-series time-course gene-expression data.

RNAi screens are frequently performed with multiple siRNAs per target gene; however, the use of population-averaged values often leads to the identification of ‘off-target’ effects as hits, since population averaged values can only monitor major variations of the phenotype such as up- or down-regulation compared to a control. In contrast, non-averaged data can indicate more diverse changes in a cell population upon treatment; thus, different siRNAs targeting the same gene should have a similar ‘on-target’ effect on the distribution profile of the measured cellular features, and consequently, these are more likely to be ‘true’ hits. The SOPRA workflow we describe here has the power to cluster all significantly altered normalized distribution profiles, identifying siRNAs with similar ‘on-target’ profiles for the same gene via a heatmap approach. Therefore, the SOPRA workflow can be used to avoid false-positive hits or ‘off-target’ effects, leading to more reliable HCS hit results and reducing time and work-intensive validation steps.

In principle, the SOPRA workflow can be used to analyze single-cell population data from various sources such as microscopy or FACS. In this study, we performed a microscopy-based high content screen of the effect of siRNA-mediated gene knockdown of selected genes taken from a published cell cycle data set from Kittler et al. [23] as an example to demonstrate the utility of the SOPRA workflow.

We were able to show that the false positive detection rate (detection of neutral controls as significantly changed) can be considerably reduced when taking into account more than one cellular feature. As described using the generated cell cycle data, we were able to demonstrate that the SOPRA workflow led to no false-positive hits among the neutral controls when at least two of the image features were taken into account. For the cell populations treated with the cell cycle inhibitors, a very high hit detection rate of 99.55% was achieved (223 of 224 cell population profiles). We also used siRNA knockdowns in this screen, which produce less significant phenotypic effects than small chemical compounds. Nevertheless, analysis of changed cell populations based on gene perturbation with siRNA using SOPRA still achieved a hit detection rate comparable to a manual FACS analysis with commercial software, which requires predetermined gating or thresholding.

Taken together, SOPRA is a novel analysis workflow that uses a unique analysis approach for non-averaged high-throughput data from cellular features based on regression analysis of normalized frequency distribution profiles of cell populations. It offers an easy-to-handle workflow and can run on hundreds of cell populations using multiple features. In particular, treated cell populations are defined as significantly changed on two measurements—the *p*-value and the RSQ-value—followed by a clustering step to identify treatments with the same normalized density profiles. The following heatmap analysis enabled us to filter out most hits that are likely to be false positives. Thus, SOPRA is a unique tool ideal for the high-content analysis of cell population data.

## Methods

### Cell cycle perturbation screen

We generated a set of screening plates consisting of siRNAs (Qiagen, Germany) targeting proteins responsible and not responsible for cell cycle progression, as well as the neutral siRNAs ‘AllStars’ and ‘Luciferase’, and wells without treatment (Mock) (Additional file 1: Fig. 1). On day one, cells were seeded in 96-well plates and transfected using Hiperfect (Qiagen, Germany). The chemical cell cycle inhibitors nocodazole and aphidicolin were added as positive controls at the described time points and concentrations. On day four, cells were fixed using 4% PFA and stained with Hoechst 33,342 (5 µg/ml, Sigma). The plates were imaged using an automated microscope (IX-81, Olympus, Germany) and analyzed using the ScanR software with an in-house image analysis assay (Additional file 3: Fig. 2).

Using a ScanR single cell export script, single-cell data were exported and downloadable from https://transfer.mpiib-berlin.mpg.de/s/AibR4AHLCR9xzDB?path=%2F. The SOPRA project description is also available from GitHub https://github.com/kppleissner/SOPRA/.

### Cell cycle FACS validation

For FACS analysis of cell cycle profiles, 1 × 10^5^ cells were seeded into each well of a 12-well plate 24 h before transfection. Cells were then transfected with Hiperfect transfection reagent (Qiagen) according to the manufacturer’s guidelines. In brief, 150 ng of specific siRNA was added to RPMI without serum and incubated with 6 µl Hiperfect in a total volume of 100 µl. After 10 to 15 min, the liposome-siRNA mixture was added to the cells with 1 ml of cell culture medium (RPMI (Gibco) supplemented with 10% fetal calf serum (FCS) (Biochrome), 2 mM glutamine, and 1 mM sodium pyruvate), to give a final siRNA concentration of 10 nM. After one day, cells were trypsinized and seeded into new 6-well plates. Three days after transfection, cells were detached from the plate with the addition of trypsin–EDTA for 5 min, spun down for 10 min at 500 × *g,* and resuspended in 0.5 ml PBS. The resuspended cells were then added to 70% ethanol for fixation and left at –20 ℃ overnight. Cells were collected by centrifugation, resuspended, rinsed in PBS, and re-collected by centrifugation. Pelleted cells were resuspended in 500 µl PBS containing a final concentration of 20 µg/ml propidium iodide and 200 µg/ml RNAse A and left in the dark for 30 min at room temperature. Cell Cycle analysis was then performed using a Becton Dickinson FACSort flow cytometer and BD CellQuest Pro Software (BD Biosciences).

### SOPRA software

The SOPRA software workflow (Fig. 1) consists of several steps: pre-processing (SOPRA1 of 4), data gathering and normalization (SOPRA 2 of 4), identification of significantly changed cell populations, and clustering of similar density profiles (SOPRA 3 of 4) and conversion of significant siRNAs into genes with their cluster membership. Furthermore, the workflow requires a variety of input files describing the experimental design and input/output folders for data.

The ‘*Single Cell Feature Files’* contain the features for every single cell measured, while the files ‘*PlateConf_LookUp’, ‘PlateList’* and ‘*ScreenLog*’ contain information about well content, plate content and flagged wells. In the first step, the data is gathered, including the flagging of wells and single objects within wells. In the next step, a plate-wise median normalization is performed, and the limits for the binning intervals are defined. Subsequently, the single objects within each binning interval (bin) are counted, and a bin-wise control-based normalization is performed. Since the derived frequency distribution profiles of measured features can be analyzed in a similar way as time curves, we employed regression analysis using R-Package maSigPro. Significantly different profiles can be identified using the calculated *p*-value, RSQ-value, and alpha-value for each sample profile. The significant profiles can be clustered using different clustering algorithms. Finally, a post-processing step (optional) can be performed to convert siRNA into gene names, cluster membership, and frequency. The SOPRA workflow is written as a Shiny application in R, enabling interactive selection of input/output folders, descriptive files characterizing plates, definitions of parameters for regression analysis etc. A detailed project description with specific instructions for how to run the workflow is available from GitHub.

### Supporting data ZIP file for 384-wells plates (single cell features)

The *384_wells_Plates_for_SOPRA.zip* file contains data based on a cell cycle screen analyzed with ScanR: High-Content Screening Station for Life Science, Olympus. The following cell features were measured: ‘Area’,’ Mean Instensity DAPI’ and ‘Total Intensity DAPI’. In general, any file in the correct format can be used for SOPRA. One file is needed for each plate or part of a plate. The folders also contain the descriptive files '*PlateConf_LookUp', 'PlateList'* and '*ScreenLog*'. https://transfer.mpiib-berlin.mpg.de/s/AibR4AHLCR9xzDB?path=%2F.

## Code availability and implementation

Source code of SOPRA shiny application (ui.R, server.R), single-cell data (96-wells plate) data for testing and SOPRA project description (folder: Manual) are freely available from GitHub https://github.com/kppleissner/SOPRA/.

## Supplementary Information

**Additional file 1: ** Screen setup includes 166 different siRNAs to target 107 genes, from which 54 had been reported to interfere with cell cycle progression in [23], known cell cycle inhibitors (A/N) in different concentrations, neutral controls (AS, L, M), as (toxic) siRNAs as a transfection control (PLK).

**Additional file 2: **A total of 166 different siRNAs to target 107 genes, from which 54 had been reported to interfere with cell cycle progression [23].

**Additional file 3: ** Automated image analysis (ScanR: High-Content Screening Station for Life Science, Olympus) for measuring Area, mean Intensity and total Intensity for each cell (=count).

**Additional file 4: ** Lists of statistically significant profiles. As expected, cell populations treated with the ‘AllStars’ or ‘Luciferase’ controls usually had high p-values and low RSQ- values. Only two (10%) and four (20%) out of 20 cellular populations treated with the neutral controls ‘Allstars’ or ‘Luciferase’, respectively, were identified to be significantly changed for at least one of the three cellular features used.

**Additional file 5: ** Comparison of SOPRA, FACS and published data. 28 profiles for each of the aphidicolin conditions A2 (4 μg/ml/24 h), A3 (2 μg/ml/12 h) and A4 (4 μg/ml/12 h), for each of the nocodazole conditions N1 (50 ng/ml/24 h), N2 (75 ng/ml/24 h), N3 (50 ng/ml/12 h) and N4 (75 ng/ml/12 h) and 27 out of 28 profiles for the aphidicolin condition A1 (2 μg/ml/24 h) were identified as significantly changed hits.

## Data Availability

The datasets and software are available via the following weblinks. Project name: SOPRA. SOPRA software, test data and project description: https://github.com/kppleissner/SOPRA/. Operating systems: Windows, Linux, MacOS Programming language: R. Other requirements: RStudio. Screen data for cell cycle progression (8 × 384 wells plates): https://transfer.mpiib-berlin.mpg.de/s/AibR4AHLCR9xzDB?path=%2F License: GNU GPL. Any restrictions to use by non-academics: None.
