# Supplementary material for: Single object profiles regression analysis (SOPRA): a novel method for analyzing high-content cell-based screens
Source: BMC Bioinformatics. 2022 Oct 21;23:440. doi: 10.1186/s12859-022-04981-8 (PMC9587636; doi:10.1186/s12859-022-04981-8)
Supplement: Supplementary file 1 — Additional file 1: Screen setup includes 166 different siRNAs to target 107 genes, from which 54 had been reported to interfere with cell cycle progression in [23], known cell cycle inhibitors (A/N) in different concentrations, neutral controls (AS, L, M), as (toxic) siRNAs as a transfection control (PLK). [file 12859_2022_4981_MOESM1_ESM.pdf]

Supplementary Figure 1

Plate1

	1	2	3	4	5	6	7	8	9	10	11	12	13	14	15	16	17	18	19	20	21	22	23	24
A																								
B		1	1	15	15	29	29	43	43	57	57	71	71	AS	M	A1	A2	N1	N2	A3	A4	N3	N4	Plk 1-1µM
C		2	2	16	16	30	30	44	44	58	58	72	72	AS	M	A1	A2	N1	N2	A3	A4	N3	N4	Plk 1-1µM
D		3	3	17	17	31	31	45	45	59	59	73	73	AS	M	A1	A2	N1	N2	A3	A4	N3	N4	Plk 1-200nM
E		4	4	18	18	32	32	46	46	60	60	74	74	AS	M	A1	A2	N1	N2	A3	A4	N3	N4	Plk 1-200nM
F		5	5	19	19	33	33	47	47	61	61	75	75	AS	L	A1	A2	N1	N2	A3	A4	N3	N4	Plk 1-40nM
G		6	6	20	20	34	34	48	48	62	62	76	76	L	L	A1	A2	N1	N2	A3	A4	N3	N4	Plk 1-40nM
H		7	7	21	21	35	35	49	49	63	63	77	77	L	L	A1	A2	N1	N2	A3	A4	N3	N4	Plk 1-8nM
I		8	8	22	22	36	36	50	50	64	64	78	78	L	L	A1	A2	N1	N2	A3	A4	N3	N4	Plk 1-8nM
J		9	9	23	23	37	37	51	51	65	65	79	79	L	L	A1	A2	N1	N2	A3	A4	N3	N4	Plk 1-1.6nM
K		10	10	24	24	38	38			66	66	80	80	L	AS	A1	A2	N1	N2	A3	A4	N3	N4	Plk 1-1.6nM
L		11	11	25	25	39	39	53	53	67	67	81	81	M	AS	A1	A2	N1	N2	A3	A4	N3	N4	
M		12	12	26	26	40	40	54	54	68	68	82	82	M	AS	A1	A2	N1	N2	A3	A4	N3	N4	
N		13	13	27	27	41	41	55	55	69	69	83	83	M	AS	A1	A2	N1	N2	A3	A4	N3	N4	
O		14	14	28	28	42	42	56	56	70	70	84	84	M	AS	A1	A2	N1	N2	A3	A4	N3	N4	
P																								

Legend for controls

AS-AllStars
L-Luci
M-mock
A1- Aphidicolin- 2ug/ml- 24hr
A2- Aphidicolin- 4ug/ml- 24hr
A3- Aphidicolin- 2ug/ml- 12hr
A4-Aphidicolin- 4ug/ml- 12hr
N1-Nocodazole- 50ng/ml- 24hr
N2-Nocodazole- 75ng/ml- 24hr
N3-Nocodazole- 50ng/ml- 12hr
N4-Nocodazole- 75ng/ml- 12hr
Test siRNA's
PLK1-transfection control
empty

Plate2

	1	2	3	4	5	6	7	8	9	10	11	12	13	14	15	16	17	18	19	20	21	22	23	24
A																								
B		85	85	99	99	113	113	127	127	141	141	155	155	AS	M	A1	A2	N1	N2	A3	A4	N3	N4	Plk 1-1µM
C		86	86	100	100	114	114	128	128	142	142	156	156	AS	M	A1	A2	N1	N2	A3	A4	N3	N4	Plk 1-1µM
D		87	87	101	101	115	115	129	129	143	143	157	157	AS	M	A1	A2	N1	N2	A3	A4	N3	N4	Plk 1-200nM
E		88	88	102	102	116	116	130	130	144	144	158	158	AS	M	A1	A2	N1	N2	A3	A4	N3	N4	Plk 1-200nM
F		89	89	103	103	117	117	131	131	145	145	159	159	AS	L	A1	A2	N1	N2	A3	A4	N3	N4	Plk 1-40nM
G		90	90	104	104	118	118	132	132	146	146	160	160	L	L	A1	A2	N1	N2	A3	A4	N3	N4	Plk 1-40nM
H		91	91	105	105	119	119	133	133	147	147	161	161	L	L	A1	A2	N1	N2	A3	A4	N3	N4	Plk 1-8nM
I		92	92	106	106	120	120	134	134	148	148	162	162	L	L	A1	A2	N1	N2	A3	A4	N3	N4	Plk 1-8nM
J		93	93	107	107	121	121	135	135	149	149	163	163	L	L	A1	A2	N1	N2	A3	A4	N3	N4	Plk 1-1.6nM
K		94	94	108	108	122	122	136	136	150	150	164	164	L	AS	A1	A2	N1	N2	A3	A4	N3	N4	Plk 1-1.6nM
L		95	95	109	109	123	123	137	137	151	151	165	165	M	AS	A1	A2	N1	N2	A3	A4	N3	N4	
M		96	96	110	110	124	124	138	138	152	152	166	166	M	AS	A1	A2	N1	N2	A3	A4	N3	N4	
N		97	97	111	111	125	125	139	139	153	153	167	167	M	AS	A1	A2	N1	N2	A3	A4	N3	N4	
O		98	98	112	112	126	126	140	140	154	154			M	AS	A1	A2	N1	N2	A3	A4	N3	N4	
P																								
