# Supplementary material for: Single object profiles regression analysis (SOPRA): a novel method for analyzing high-content cell-based screens
Source: BMC Bioinformatics. 2022 Oct 21;23:440. doi: 10.1186/s12859-022-04981-8 (PMC9587636; doi:10.1186/s12859-022-04981-8)
Supplement: Supplementary file 2 — Additional file 2: A total of 166 different siRNAs to target 107 genes, from which 54 had been reported to interfere with cell cycle progression [23]. [file 12859_2022_4981_MOESM2_ESM.pdf]

Supplementary Table 1

GeneSymbol	**Gene ID**	# of siRNA tested
ABI1	10006	2
ABI2	10152	2
ACTR3	10096	1
ADAM17	6868	1
AKAP6	9472	1
ALDH1A2	8854	1
ANP32A	8125	2
AP1B1	162	2
APAF1	317	2
ARF4	378	2
ARFGAP1	55738	2
ATF2	1386	2
ATG3	64422	2
ATG4D	84971	1
ATG5	9474	2
ATG9A	79065	1
ATR	545	2
AVEN	57099	1
AXIN2	8313	1
BAG1	578	2
BARD1	580	1
BAT1	7919	2
BCAT1	586	2
BCL10	8915	1
BCL2	596	2
BCL2L1	598	2
BCL2L11	10018	2
BCL3	602	1
BCR	613	1
BET1	10282	2
BET1L	51272	2
BID	637	2
BIRC2	329	2
BIRC3	330	1
BIRC4	331	2
BIRC5	332	2
BMF	90427	2
BNIP1	662	1
CALR	811	1
CAPN1	823	1
CARD10	29775	1
CASP1	834	2
CASP14	23581	2

Legend	
(white)	Hits
(shaded)	Non-Hits

CASP2	835	1
CASP3	836	2
CASP5	838	2
CASP7	840	1
CAV1	857	2
CBL	867	2
CCNE1	898	1
CCT2	10576	1
CD40LG	959	1
CD44	960	1
CD69	969	1
CDC2	983	2
CHEK1	1111	1
CLK1	1195	2
CLK2	1196	2
CNN3	1266	1
COPB	1315	1
CREB1	1385	2
CARHSP1	23589	2
CSK	1445	2
CTNNA1	1495	2
DDX1	1653	1
EIF3S4	8666	2
FTL	2512	1
GABARAPL1	23710	1
HNRPH1	3187	2
HNRPK	3190	1
HNRPM	4670	1
IK	3550	1
INCENP	3619	2
KIF20A	10112	1
KIF3A	11127	1
KIF5B	3799	1
LIMK1	3984	2
MCL1	4170	1
MYC	4609	2
NCL	4691	3
NEK7	140609	2
NXF1	10482	2
PHB	5245	2
PICK1	9463	2
PLCG2	5336	1
PLK1	5347	2
PRDX3	10935	2
PRKD3	23683	1
PRPF8	10594	2
PSMA3	5684	2
PSMA7	5688	1

PSMB1	5689	2
PSMC3	5702	1
PSMC4	5704	1
PSMC5	5705	2
PSMD7	5713	1
RBBP4	5928	1
ROCK1	6093	1
RPLP0	6175	2
SERPINB9	5272	2
SF3A1	10291	2
SNRPA1	6627	1
TGFBR1	7046	1
TNFRSF21	27242	1
TSG101	7251	1
WEE1	7465	2
YWHAZ	7534	2
