# Supplementary figures and images for: Single object profiles regression analysis (SOPRA): a novel method for analyzing high-content cell-based screens
Source: BMC Bioinformatics. 2022 Oct 21;23:440. doi: 10.1186/s12859-022-04981-8 (PMC9587636; doi:10.1186/s12859-022-04981-8)

# Supplementary Figure 2

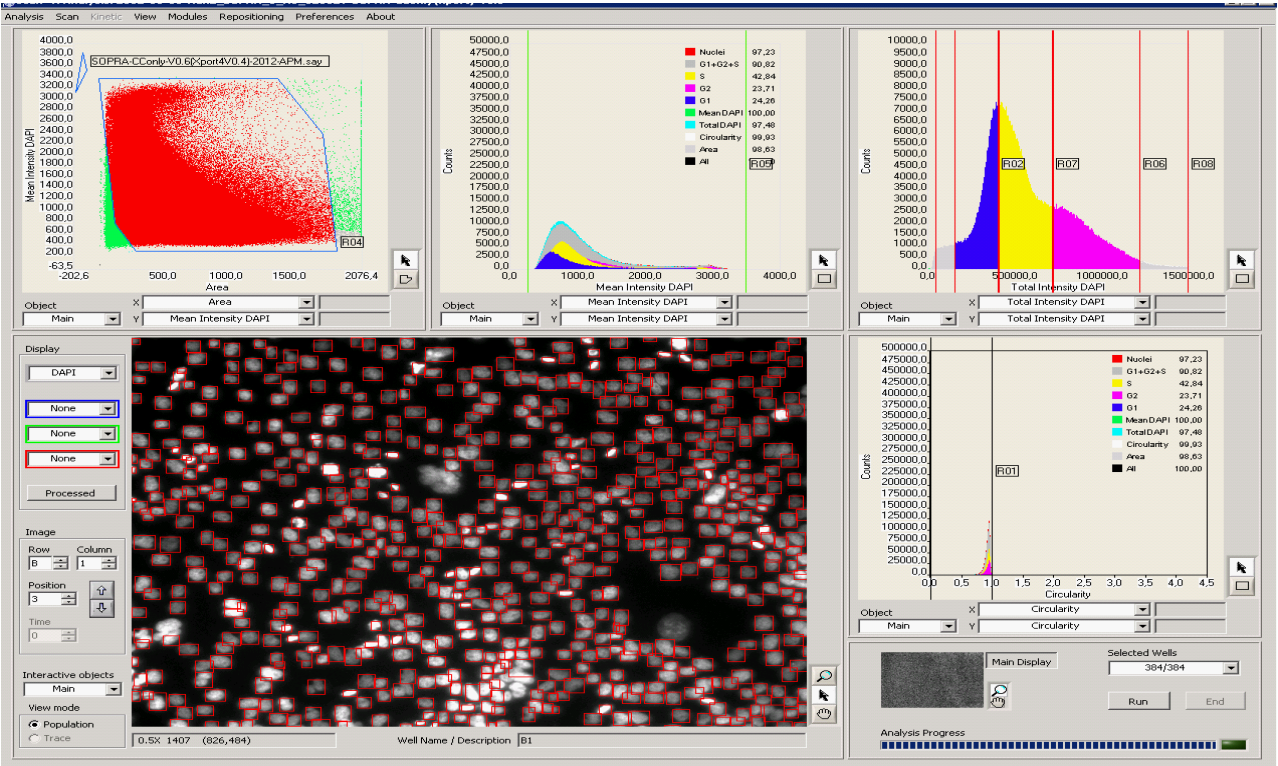

Supplement: Supplementary file 3 — Additional file 3: Automated image analysis (ScanR: High-Content Screening Station for Life Science, Olympus) for measuring Area, mean Intensity and total Intensity for each cell (=count). [file 12859_2022_4981_MOESM3_ESM.pdf]
