# Supplementary material for: Single object profiles regression analysis (SOPRA): a novel method for analyzing high-content cell-based screens
Source: BMC Bioinformatics. 2022 Oct 21;23:440. doi: 10.1186/s12859-022-04981-8 (PMC9587636; doi:10.1186/s12859-022-04981-8)

# Supplementary Figure 3

| Treatment | | | % |
|---|---|---|---|
| ***A1*** | 27 | 28 | 96.43 |
| ***A2*** | 28 | 28 | 100.00 |
| ***A3*** | 28 | 28 | 100.00 |
| ***A4*** | 28 | 28 | 100.00 |
| ***N1*** | 28 | 28 | 100.00 |
| ***N2*** | 28 | 28 | 100.00 |
| ***N3*** | 28 | 28 | 100.00 |
| ***N4*** | 28 | 28 | 100.00 |
| ***Luci*** | 4 | 20 | 20.00 |
| ***Mock*** | 3 | 18 | 16.67 |
| ***Allstars*** | 2 | 20 | 10.00 |

| | *Number of ..* | | *Per cent of ..* |
|---|---|---|---|
| | *..genes as hits or nonhits* | *..genes validated by at least one other method* | |
| SOPRA hits | 35 | 29 | 83% |
| SOPRA nonhits | 9 | 7 | 78% |
| FACS hits | 33 | 29 | 88% |
| FACS nonhits | 11 | 9 | 82% |
| Paper hits | 19 | 18 | 95% |
| Paper nonhits | 25 | 12 | 48% |

B hits:

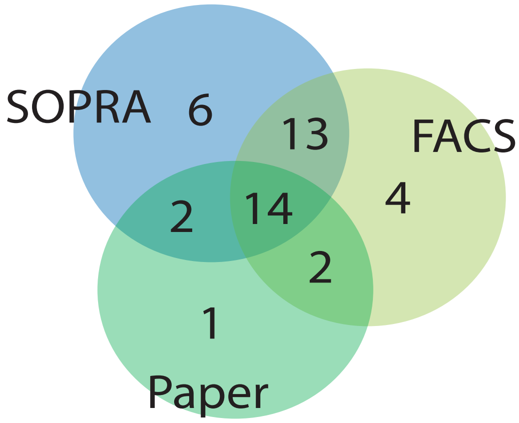


nonhits:

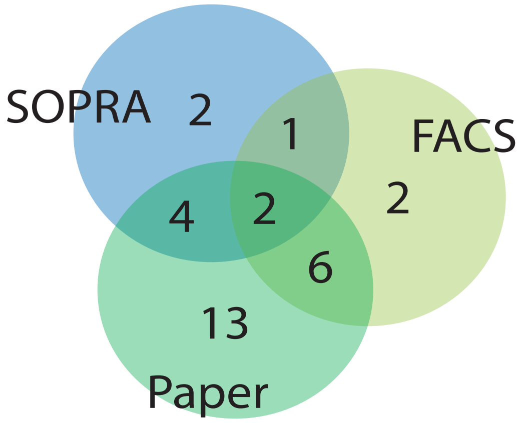

Supplement: Supplementary file 5 — Additional file 5: Comparison of SOPRA, FACS and published data. 28 profiles for each of the aphidicolin conditions A2 (4 μg/ml/24 h), A3 (2 μg/ml/12 h) and A4 (4 μg/ml/12 h), for each of the nocodazole conditions N1 (50 ng/ml/24 h), N2 (75 ng/ml/24 h), N3 (50 ng/ml/12 h) and N4 (75 ng/ml/12 h) and 27 out of 28 profiles for the aphidicolin condition A1 (2 μg/ml/24 h) were identified as significantly changed hits. [file 12859_2022_4981_MOESM5_ESM.pdf]
